# Is it possible to contribute to the recovery of European grayling (*Thymallus thymallus*, Salmonidae) populations by stocking cultured brood fish in the pre‐spawning period?

**DOI:** 10.1111/jfb.16071

**Published:** 2025-02-12

**Authors:** Mladen Avramović, Jan Turek, Tomáš Randák

**Affiliations:** ^1^ Faculty of Fisheries and Protection of Waters, South Bohemian Research Center of Aquaculture and Biodiversity of Hydrocenoses University of South Bohemia in České Budějovice Vodňany Czech Republic

**Keywords:** adaptability, brood stock, electrofishing, fishery management, spawning success

## Abstract

European grayling populations have declined significantly in their central range, prompting numerous stocking programs with reared fish that did not bring desirable population prosperity. This study evaluated the effectiveness of stocking long‐reared grayling brood fish before their spawning period. It focused on monitoring the presence of juveniles coming from their natural spawn in the stream and on recapture rates and growth parameters of stocked fish to estimate their adaptability in a natural river environment. The results revealed that the recapture rate of the stocked brood fish was notably low, with significant increases only in length growth but a decreases in condition factor, suggesting poor adaptability in the wild. The limited number of young‐of‐year grayling from natural spawning further indicated low reproductive success. These results highlight the inadequacies of this stocking approach to strengthen the wild grayling population. We suggest alternative strategies, such as using younger stock and implementing protective measures like catch‐and‐release, which may improve conservation efforts and enhance the success of grayling stocking programs.

Fish stocking is often a key action in fishery management, compensating for recruitment losses or extinction threats caused by overfishing, predation, or habitat degradation (Cowx, 1994; Einum & Fleming, 2001; Simonović et al., 2014). In the Czech Republic, local management of grayling is based on stocking with 1+ and 2+ fish (Horká et al., 2015; Turek et al., 2010, 2012, 2018), yet these efforts have not secured a self‐sustaining population, and the decline of grayling population is continuing. Hypothetically, the brood fish represent a valuable source for the enhancement of population by natural spawning and creating a new young population class of wild grayling, but such practice of introducing reared broodstock wild salmonid for strengthening the structure of the wild local population is rarely applied. While stocking with fry has shown some success (Avramović et al., 2024; Carlstein, 1997; Czerniawski et al., 2015; Romakkaniemi, 2008), stocking brood fish raises concerns due to long‐term rearing that reduces adaptability (Araki et al., 2007; Einum & Fleming, 2001; Fraser et al., 2011). As grayling age, they tend to inhabit deeper and faster waters (Degerman et al., 2000; Riley & Pawson, 2010) and shift their diet towards benthic feeding (Dahl, 1962). This behavior is supposed to reduce their exposure to bird predators, a significant threat to grayling populations (Jepsen et al., 2018). Also, older grayling are expected to better cope with the water current (Auer et al., 2023) and have increased resilience to disease (Carraro et al., 2017; Okamura et al., 2011) than the grayling 0+ category.

The local situation in the Blanice River, Czech Republic, aligns with general predictions, as previous restocking efforts focused on releasing 1+‐ and 2+‐year‐old grayling over an extended period have proven ineffective. These efforts failed to lead to the long‐term enhancement of the Blanice grayling population, mainly due to the lack of successful reproduction. Given these challenges, this study aimed to test whether stocking brood fish approximately 1 month before spawning can help to reintroduce grayling by their instinctive natural spawning. We hypothesized that while the stocked fish may not survive long‐term, their offspring could establish a new generation, potentially restoring a functional grayling population.

The experiment took place in the upper section of the Blanice River (South Bohemia region, Czech Republic), within the special fishing ground of the Faculty of Fisheries and Protection of Waters (FFPW), University of South Bohemia. The fishing regulation for grayling is catch and release (C&R). On March 11, 2020, about a month before supposed grayling spawning, electrofishing survey of 5 km was performed and confirmed the presence of two matured grayling distanced from each other. Within this 5‐km part we chose three stretches (each 150 m) for stocking, positioned far from the observed wild grayling. Grayling brood fish (3 years old) farmed in a small pond located on the Živny stream, a tributary of the Blanice River, were collected. A total of 150 mature grayling (50:50 sex ratio) were randomly selected, measured, weighed, and tagged with micro‐Passive Integrated Transponder (PIT) tags (Loligo FDX‐B, 7 × 1.35 mm). Tags were injected intramuscularly near the dorsal fin, followed by disinfectant (KMnO_4_) treatment. Fish were then divided into three groups of 50 (25 males, 25 females) and held in a hatchery supplied by Blanice water to minimize stress before release. On March 12, each group was released into the upper part of the long‐term monitored stretches. After 7 months (October 12, 2020), electrofishing was conducted over 5 km of Blanice River, including all three long‐term monitored stretches, to assess the presence of young‐of‐the‐year grayling and tagged brood fish. Recaptured brood fishes were identified with a PIT‐tag reader (Trovan, ARE H5), measured, and weighed. Captured progeny were also measured. Data were analyzed using GraphPad Prism (v10.0). A paired *t*‐test compared biometric measures (standard length, weight, and Fulton's condition factor) of the same individuals before stocking and after recapture. Chi‐squared tests evaluated differences in migration from the release site, site fidelity, and migration direction (upstream or downstream). Site fidelity, defined as fish being recaptured at the release site, was compared to the observed number of those recaptured out of the release site (migrants).

Additionally, we compared the dispersal directions (upstream and downstream) among those migrants. Significance was set at *p* < 0.05. This study was conducted following the ethical guidelines of the Czech Republic and received approval from the relevant ethics committee. The treatment and welfare of fish fully adhered to the legal requirements in the Czech Republic (§ 7 Law No. 114/1992 on The Protection of Nature and Landscape and § 6, 7, 9, and 10 Regulation No. 419/2012 on the Care, Breeding, and Use of Experimental Animals).

Post‐stocking electrofishing recaptured 11 tagged brood fish across multiple sites, reflecting a low recapture rate of 7.3% after 7 months. No significant changes between stocked and recaptured individuals were observed in weight (W), standard length (SL) increased (*p* < 0.03, *n* = 11) while their Fulton's condition factor significantly declined (*p* < 0.01, n = 11) (Figure 1). Migration was notably higher than site fidelity (*p* = 0.02), with no significant difference between upstream and downstream movement (*p* = 0.11). Additionally, 11 grayling young of the year (YOY) individuals were recaptured, showing a mean SL of 120.8 ± 9.8 mm and a mean weight of 20.9 ± 4.3 g.

**FIGURE 1:**
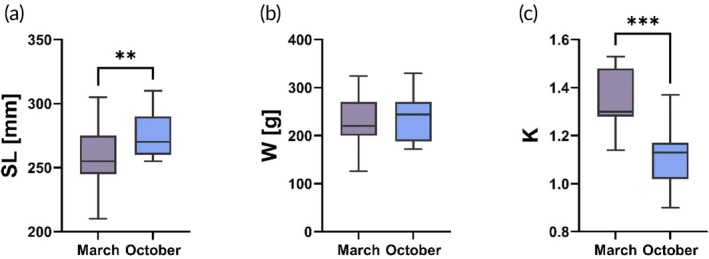
Biometric parameters of European grayling brood fish stocked in March 2020 and recaptured in October 2020. The graph shows a significant increase in standard length (a), no change in weight (b), and a significant decrease in Fulton's condition factor (c). Asterisks denote significant differences (**p < 0.01 and ***p < 0.001).

The low recapture rate signals poor stocking efficiency, notably lower than in prior attempts with younger grayling in the same river stretches (Turek et al., 2010, 2012, 2018). The significant drop in condition factor suggests reduced fitness, consistent with findings on the adverse impacts of long‐term captivity on salmonids (Araki et al., 2007; Avramović et al., 2024; Einum & Fleming, 2001). Despite expectations that larger brood fish would cope better with the flowing conditions in the wild (Auer et al., 2023), their significant post‐stocking migrations align with previous studies on reared grayling that exhibited similar migratory behavior (Horká et al., 2015; Thorfve, 2002; Thorfve & Carlstein, 1998). Since it is hard to document apparent sub‐lethal bird predation on fish, it might have taken a considerable share in the overall loss of stocked fish (Figure 2), implying low predatory response of long‐reared grayling. The recorded occurrence of only 11 YOY grayling individuals originating very likely from the natural spawning of introduced brood fish indicates reduced natural reproduction abilities induced by long‐rearing time (Araki et al., 2007). We maintained the minimal probability of YOY individuals drifting into our monitored area from the short, non‐monitored stretch upstream, which still aligns with our finding of reproductive incapability of long‐reared grayling in the wild. Alternatively, the environmental disruption of spawning grounds affected spawning efficiency. Such disruptions are expected in fragmented rivers like the Blanice, where weirs can hinder the natural transport of suitable spawning sediment (Hauer et al., 2011; Schmutz & Moog, 2018). However, the great length and weight growth of these young fish show strong adaptability. This further suggests stocking materials should be introduced at the earliest possible age (Persat et al., 2016) to closely mimic the natural development process. Additionally, it implies trying more comprehensive stocking practices than the simple release of reared fish to achieve this aim, such as the plantation of fertilized eggs in the in‐stream incubators. These practices aimed to exclude artificial conditions during the incubation that can alter the behavior and emergence timing of grayling (Bardonnet & Gaudin, 1990), while natural selection would affect these early stages and increase the elimination of non‐adaptive larvae (Vincenzi et al., 2012). Given the low efficiency and high resource demands, the stocking strategy with grayling brood fish appears questionable, raising concerns over its practical and ecological impact (Hunt & Jones, 2018; Simonović et al., 2014). Based on our results, we recommend that the presented restocking practice should be clearly dispelled from the consideration of strengthening the local grayling population. Furthermore, in fishing grounds where grayling is not under C&R regulations, we assume that a significant increase in the minimum catching size can also serve as an important protective measure for wild grayling brood fish, which play a pivotal role in population renewal. Accordingly, such an increase (from 30 to 40 cm) has already decreased the number of grayling killed by anglers (Lyach & Remr, 2019). It should be noted that, in parallel with measures in fishery management, it is essential to address the effective regulation of overabundant fish predators, as well as foster awareness and commitment from anglers, the angling industry, and managers to engage in responsible actions that promote sustainability (Cooke et al., 2019) and mitigate overfishing risks intensified by environmental changes (Ayllón et al., 2025). Lastly, in areas where grayling populations are critically low, restocking is crucial and we suggest the need to shift stocking practices towards earlier life stages and explore alternative release methods to achieve better outcomes.

**FIGURE 2:**
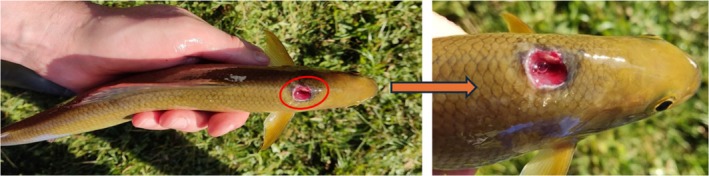
A European grayling (*Thymallus thymallus*) brood fish, recaptured 7 months after stocking, shows a severe scar caused by a predatory bird.

## AUTHOR CONTRIBUTIONS

Conceptualisation, Mladen Avramović and Tomáš Randák; Formal Analysis, Mladen Avramović, Jan Turek; Investigation, all authors; Resources, Tomáš Randák; Writing ‐ Mladen Avramović; Writing – Review and Editing, Mladen Avramović, Tomáš Randák and Jan Turek; Visualisation, Mladen Avramović; Project Administration and Funding Acquisition, Tomáš Randák. All authors reviewed the manuscript.
